# Delayed Administration of IGFBP7 Improved Bone Defect Healing via ZO‐1 Dependent Vessel Stabilization

**DOI:** 10.1002/advs.202406965

**Published:** 2024-12-19

**Authors:** Shiyu Sun, Yao Li, Yuman Li, Yuting Niu, Zhewen Hu, Chenyu Deng, Yiming Chen, Bo Hu, Ying Huang, Xuliang Deng

**Affiliations:** ^1^ Department of General Dentistry Peking University School and Hospital of Stomatology Beijing 100081 P. R. China; ^2^ Department of Geriatric Dentistry Peking University School and Hospital of Stomatology Beijing 100081 P. R. China; ^3^ Department of Orthodontics Peking University School and Hospital of Stomatology Beijing 100081 China; ^4^ National Clinical Research Center for Oral Diseases & National Engineering Research Center of Oral Biomaterials and Digital Medical Devices& Beijing Key Laboratory of Digital Stomatology & NHC Key Laboratory of Digital Stomatology & NMPA Key Laboratory for Dental Materials Beijing 100081 P. R. China

**Keywords:** vessel stabilization, IGFBP7, bone defect repair, endothelial tight junctions, Zonula Occluden‐1, AT1001

## Abstract

The vascular response following injury is pivotal for successful bone‐defect repair but constitutes a major hurdle in the field of regenerative medicine. Throughout this process, vessel stabilization is crucial to provide an adequate nutrient supply and facilitate efficient waste removal. Therefore, this study investigated whether promoting vascular stabilization improves bone defect repair outcomes. The findings show that insulin‐like growth factor‐binding protein (IGFBP) 7 exhibits a novel biological function in attenuating vascular permeability and enhancing vascular wall integrity. The potential underlying mechanism involves the up‐regulation of insulin‐like growth factor 1 receptor (IGF1R) expression by IGFBP7 on endothelial cell membrane, followed by activation of the downstream PI3K/AKT signaling pathway and upregulated expression of the tight junction protein zonula occludens‐1 (ZO‐1). IGFBP7 delayed administration in mice with cranial defects significantly improved bone defect healing by increasing ZO‐1 and CD31 co‐localization within vessel walls and optimizing the perfusion function of the final vascular network. Furthermore, the application of the typical tight junction regulator AT1001 effectively promoted ZO‐1‐dependent vascular stabilization and facilitated bone defect repair. This study presents a new approach to enhance bone defect healing via vascular stabilization‐targeted interventions and significantly advances the understanding of the complex interplay between osteogenesis and angiogenesis in bone defect healing.

## Graphical abstract

This research uncovers a new biological function of IGFBP7 in promoting vascular stability by upregulating ZO‐1 protein expression within endothelial tight junctions. The findings thus demonstrate the ability of IGFBP7 to expedite bone defect healing while concurrently enhancing blood vessel stabilization, providing continuous support for tissue repair. This work proposes vascular stabilization as an innovative intervention target for bone defect repair.

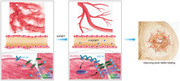

## 1 Introduction

Nascent blood vessels that are formed by angiogenic sprouting are widely considered a preliminary version of the vascular network. These nascent blood vessels undergo extensive remodeling during a crucial angiogenic stage known as vessel stabilization that follows sprouting angiogenesis to establish a structurally stable and functional vasculature.^[^ ^1^, ^2^ ^]^ Vessel stabilization is a biological process following immature vasculature formation that optimizes vessel wall functionality and subsequently establishes steady blood flow.^[^ ^3^ ^]^ Fractures, tumor resections, infections, deformities, sports injuries, and other physical traumas lead to bone defects, which pose an intractable challenge in clinical orthopedic therapy and are a focal point of current research because of the high risk of delayed or failed healing.^[^ ^4^ ^]^ The healing of bone defects critically depends on robust vascular perfusion within the compromised region, which is essential for optimizing cellular metabolism and facilitating subsequent regenerative outcomes. Thus, sustaining an adequate blood supply to the injured site is crucial for establishing the necessary milieu for cellular activity, defect repair, and tissue regeneration.^[^ ^5^, ^6^ ^]^ Hence, this study aimed to determine whether promoting vessel stabilization improves bone defect repair outcomes.

Insulin‐like growth factor (IGF)‐binding protein 7 (IGFBP7) is a secreted protein that is implicated in sprouting angiogenesis and vessel remodeling in various physiological and pathological processes such as tumor and tissue regeneration.^[^ ^7^, ^8^, ^9^ ^]^ Breast cancer studies have shown that xenografted tumors exhibit decreased angiogenesis following tail vein injections of recombinant IGFBP7.^[^ ^10^, ^11^ ^]^ IGFBP7 overexpression in hepatocellular carcinoma (HCC) cells inhibits in vitro cell proliferation, in vivo tumor growth, and angiogenesis, thereby functioning as a novel putative tumor suppressor.^[^ ^8^ ^]^ Additionally, IGFBP7 is crucial for tissue regeneration.^[^ ^12^ ^]^ Adipose‐derived stem cells secrete IGFBP7, which regulates angiogenesis and prevents keloid formation.^[^ ^13^ ^]^ However, the effects of IGFBP7 on the regulation of vessel stabilization and its underlying mechanisms remain unclear.

Reinforcement of cell–cell interactions is a key factor in the anastomosis of vascular loops, which is a crucial step in vessel stabilization.^[^ ^14^ ^]^ Vessel stabilization involves the enhancement of the integrity of newly formed blood vessels. Tight junctions play a crucial role in this process. Cell–cell interactions between neighboring endothelial cells (ECs) are composed of tight junctions (TJs), adherent junctions (AJs), and gap junctions.^[^ ^14^ ^]^ Among these, tight junctions form a continuous intercellular barrier between ECs and separate tissue spaces while regulating the selective movement of solutes across the endothelium.^[^ ^15^, ^16^, ^17^ ^]^ The major components of tight junctions include zonula occluden‐1 (ZO‐1), claudin family members, and occludins.^[^ ^18^ ^]^ ZO‐1 interacts with transmembrane proteins such as occludins and claudins and links them to the cytoskeleton.^[^ ^19^ ^]^ Therefore, we investigated whether IGFBP7 regulates vascular stability by regulating the tight junction protein ZO‐1 to improve bone defect healing outcomes.

In this study, we aimed to determine the mechanism underlying the contribution of IGFBP7 to vessel stabilization with regard to the regulation of the tight junction protein ZO‐1 during delayed stages of bone defect healing. Additionally, we aimed to explore possible therapeutic interventions in bone defect healing via the enhanced formation of intercellular tight junctions. We believe that this study would enhance the existing knowledge on the coupling relationship of osteogenesis–angiogenesis interactions in bone defect healing. Furthermore, we aimed to highlight the value of TJ regulators in clinical application, particularly for modulating the stabilization of neovascular networks, to present promising and broad application prospects for vascular stabilization in the field of tissue regeneration.

## 2 Results

### 2.1 IGFBP7 Enhances the Integrity of the Vascular Wall Barrier by Targeting Tight Junction Protein ZO‐1

**Figure 1:**
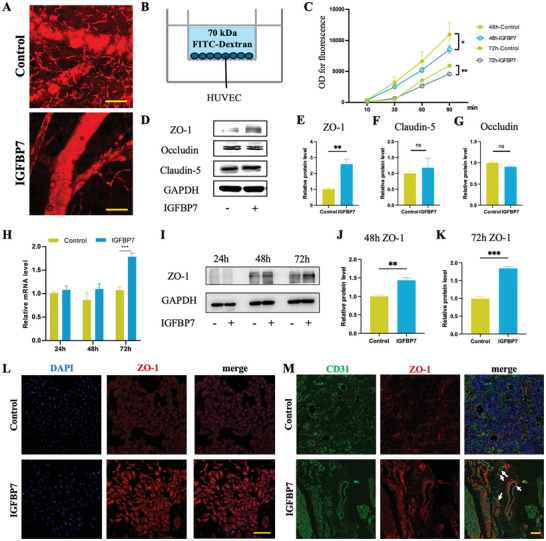
Insulin‐like growth factor (IGF)‐binding protein 7 (IGFBP7) enhances the integrity of the vascular wall barrier by targeting tight junction protein zonula occludens‐1 (ZO‐1). A) Representative images of subcutaneous transplantation of human umbilical vein endothelial cells (HUVECs) in nude mice with or without IGFBP7 (160 ng m^−1^) for 6 days, as observed under a two‐photon microscope. Scale bar: 50 µm. B) Schematic illustration of the in vitro vascular permeability assessment. C) Permeability of FITC‐dextran through the HUVEC monolayer significantly decreased after stimulation with IGFBP7 (160 ng mL^−1^) for 48 and 72 h. D) Western blotting analysis showed that ZO‐1 expression significantly increased upon treatment with IGFBP7 (160 ng mL^−1^), whereas claudin‐5 and occludin expression levels showed no significant difference. GAPDH was used as the internal control. E–G) Quantitative analysis of ZO‐1, claudin‐5, and occludin using western blotting analysis (data presented in Figure D). (H) Real‐time polymerase chain reaction (RT‐PCR) results showed that ZO‐1 mRNA expression increased in HUVECs after IGFBP7 stimulation for 24, 48, and 72 h. I) Western blotting analysis showed that exposure to IGFBP7 for 48 and 72 h significantly upregulated ZO‐1 protein expression compared with that of the control group. J) Quantitative analysis of the western blotting analysis results presented in Figure F. K) Representative images of immunofluorescence (IF) staining of ZO‐1 in HUVECs cultured with or without IGFBP7 stimulation at 160 ng mL^−1^. Scale bar: 100 µm. (L) Representative images of IF staining of subcutaneous tumor sections observed using a laser confocal microscope. Scale bar: 50 µm. All data in the figure are presented as mean ± SEM of three independent experiments, unless otherwise stated (^*^ *p* < 0.05, ^**^ *p* < 0.01, and ^***^ *p* < 0.001).

To determine the role of IGFBP7 in vascular wall integrity, we performed in vivo subcutaneous blood vessel regeneration assays (Figure S1, Supporting Information). Observations of FITC‐dextran leakage under a two‐photon confocal microscope showed that IGFBP7‐treated blood vessel walls exhibited high completeness and smoothness (Figure 1A). Conversely, the control group blood vessel walls exhibited roughness and intermittent shadows, indicative of significant increase in vascular permeability. Next, we cultured human umbilical vein endothelial cells (HUVECs) in a transwell chamber to construct a cell monolayer, as shown in Figure 1B. After culturing for 48 and 72 h, the leakage of FITC‐dextran in the IGFBP7‐treated group was significantly lower than that of the control group, suggesting that IGFBP7 reduced cell layer permeability and promoted barrier function in the HUVECs (Figure 1C; Figure S2, Supporting Information). These findings confirmed that IGFBP7 enhanced the integrity of the vascular wall barrier and attenuated vascular permeability.

Tight junctions form a continuous intercellular barrier between epithelial cells, separate tissue spaces, and modulate permeability across the EC layer.^[^ ^20^, ^21^ ^]^ The major components of tight junctions include zonula occluden‐1 (ZO‐1), claudins, and occludins. ZO‐1 is a cytoplasmic protein, whereas occludin and claudin‐5 are transmembrane proteins located in the plasma membrane, and they bind to ZO‐1 within the cell.^[^ ^22^ ^]^ Western blotting analysis was performed to determine the type of tight junction‐related protein that was affected by IGFBP7. The results showed that ZO‐1 expression levels significantly increased upon treatment with IGFBP7 (160 ng. mL^−1^), whereas claudin‐5 and occludin expression showed no significant difference (Figure 1D–G). This suggests that IGFBP7 specifically upregulates ZO‐1 expression rather than that of claudin‐5 and occludin. Next, qPCR and western blotting analyses were performed to verify the regulatory effects of IGFBP7 on ZO‐1. HUVECs in the treatment group showed increased ZO‐1 mRNA and protein expression levels than that observed in the control group after IGFBP7 treatment for 24, 48, and 72 h (Figure 1H–J). Immunofluorescent (IF) staining directly corroborated the IGFBP7‐mediated upregulation of ZO‐1 expression in ECs (Figure 1K). In total, these results confirmed that IGFBP7 promotes barrier function in HUVECs single‐cell layer and that the tight junction protein ZO‐1 is a probable downstream regulatory target of IGFBP7.

IF staining of subcutaneous tumor sections was performed to confirm the regulatory effects of IGFBP7 on ZO‐1 in vivo. The sections showed stronger co‐localization of ZO‐1 with CD31 in vessels following IGFBP7 treatment compared with that observed in the control group. This indicated the extensive presence of tight junction proteins within the vascular wall (Figure 1L). These findings further validate the effect of IGFBP7 in enhancing blood vessel stabilization through the promotion of ZO‐1 expression in vivo.

Taken together, these results validate the newly discovered biological function of IGFBP7 it enhances vascular wall barrier integrity by targeting ZO‐1.

### 2.2 IGFBP7 Promotes ZO‐1 Expression by Activating IGF1R‐PI3K/AKT Signaling Pathway

**Figure 2:**
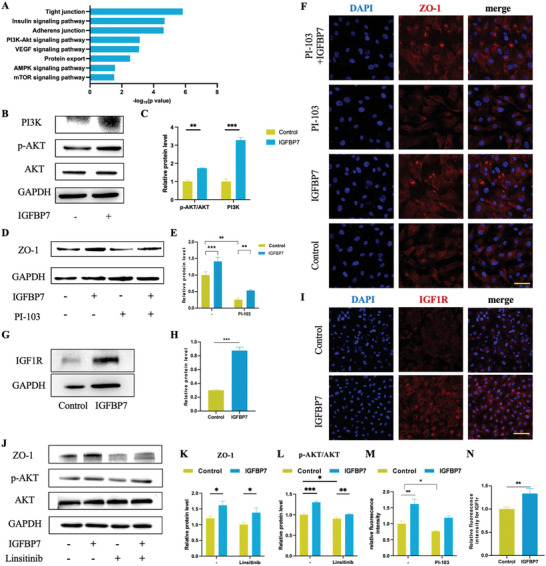
IGFBP7 promotes ZO‐1 expression through activation of the IGF1R‐PI3K/AKT signaling pathway. A) Functional enrichment analysis of differentially expressed genes in HUVECs cocultured with or without IGFBP7 (160 ng mL^−1^) performed using Kyoto Encyclopedia of Genes and Genomes (KEGG) pathway analysis. B) Western blotting analysis showed that p‐AKT, AKT, and PI3K expression significantly increased with IGFBP7 treatment (160 ng mL^−1^). C) Quantitative analysis of western blotting analysis results presented in Figure B. D) Western blotting analysis showed that ZO‐1 expression significantly decreased upon treatment with PI‐103 (10 µм), which is a PI3K signaling inhibitor. E) Quantitative analysis of western blotting analysis results presented in Figure D. F) Representative images of immunofluorescence staining of HUVECs cultured with or without IGFBP7 (160 ng mL^−1^), accompanied with or without PI‐103 (10 µм). G) Western blotting analysis showed that IGF1R expression significantly increased after treatment with IGFBP7 (160 ng mL^−1^). H) Quantitative analysis of western blotting analysis results presented in Figure G. I) Representative images of IF staining of IGF1R from HUVECs cultured with or without IGFBP7 stimulation (160 ng mL^−1^). J) Western blotting analysis showed that ZO‐1, p‐AKT, and AKT expression significantly decreased upon treatment with Linsitinib (1 µм), which was used as an IGF1R inhibitor, but subsequently increased upon treatment with Linsitinib and IGFBP7 (160 ng mL^−1^). K,L) Western blotting quantitative analysis of data presented in Figure J representing ZO‐1 and p‐AKT. M) Quantitative analysis of IF staining results presented in Figure F. N) Quantitative analysis of IF staining results presented in Figure I. All data in this figure are presented as mean ± SEM of the three independent experiments unless otherwise stated (^*^ *p* < 0.05, ^**^ *p* < 0.01, and ^***^ *p* < 0.001).

To elucidate the mechanisms underlying IGFBP7‐mediated regulation of ZO‐1 expression, we performed tandem mass tag (TMT) quantitative proteomic analysis to compare the protein expression profiles of HUVECs with and without IGFBP7 treatment (Figure S3A, Supporting Information). The results of the Kyoto Encyclopedia of Genes and Genomes (KEGG) pathway analysis showed that the upregulated differentially expressed proteins in the IGFBP7 treated group were primarily associated with tight junctions and the insulin signaling pathway, among other pathways, unlike that observed for the control group (Figure 2A). Phosphoinositide 3‐kinase (PI3K) and protein kinase B (AKT) were identified as key molecules involved in the insulin signaling pathway. Therefore, we speculated that the PI3K/AKT pathway may be involved in subsequent intracellular molecular events following IGFBP7 stimulation.

We performed western blotting analysis to further investigate whether IGFBP7 promotes ZO‐1 expression through the PI3K/AKT signaling pathway. The results showed that IGFBP7 treatment significantly enhanced PI3K and phosphorylated AKT (p‐AKT) expression (Figure 2B,C). Furthermore, PI‐103 (PI3K inhibitor)‐treated HUVECs consistently demonstrated significantly reduced ZO‐1 expression in both western blotting (Figure 2D,E) and IF (Figure 2F,M) analyses. However, IGFBP7 treatment significantly reversed the inhibitory effects of PI‐103 on ZO‐1 expression. These findings emphasize the role of the PI3K/AKT signaling pathway in promoting downstream ZO‐1 expression.

Insulin‐like growth factor 1 receptor (IGF1R) is a transmembrane receptor tyrosine kinase that is responsible for mediating IGF bioactivity. Bioinformatic analysis has shown that the IGF1R may be a potential candidate transmembrane structure that mediates extracellular‐to‐intracellular signal transduction across the cell membrane (Figure S3B, Supporting Information). Western blotting analysis (Figure 2G,H) and IF detection (Figure 2I,N) confirmed the upregulated expression of the IGF1R in HUVECs upon IGFBP7 activation. Flow cytometry experiments further corroborated IGFBP7‐mediated promotion of IGF1R expression on the cell membrane (Figure S3C,D, Supporting Information). Furthermore, IGF1R blocking with Linsitinib downregulated p‐AKT and ZO‐1 expression, whereas IGFBP7 treatment significantly reversed these inhibitory effects (Figure 2J–L). These results confirmed that IGFBP7 promotes ZO‐1 expression by activating the IGF1R‐PI3K/AKT signaling pathway.

### 2.3 IGFBP7 Regulates the Stability of Vascular Networks to Promoting Bone‐Defect Healing In Vivo

**Figure 3:**
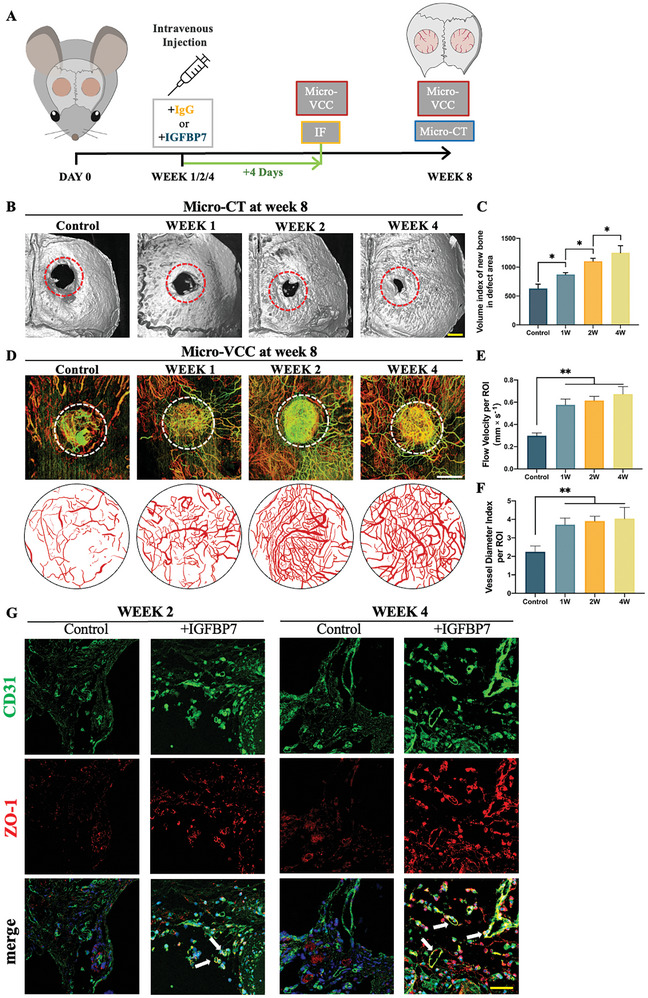
IGFBP7 regulates the stability of vascular networks and promotes bone defect healing in vivo. A) Schematic illustration of IGFBP7/IgG administration in mice and timeline of IF, micro‐CT, and micro‐vascular microcirculation (VCC) experiments. B) Representative micro‐CT 3D reconstruction images demonstrating regeneration of new bone at 8 weeks after cranial bone defection surgery. Scale bar: 1 mm. C) Relative volume index of new bone in the defect area quantified using the Inveon Research Workplace software and exhibiting a positive correlation with the timing of IGFBP7 administration; *n* = 6. D) Representative micro‐VCC scanning showing vessel networks within the cranial defect area 4 days after IGFBP7 stimulation at different time points. The composite masked map of each image using Pyoct 7.0 software is presented below. It shows reduced blood vessel density and increased vessel diameter index with time. Scale bar: 800 µm. E) Flow velocity information per area of interest (ROI) generated in “FlowVel” mode and exported as numerical values in unit mm s^−1^; *n* = 6. F) Vessel diameter index per ROI for each group in Figure D quantified using vResolve software; *n* = 6. G) Representative images of IF staining of CD31 and ZO‐1 at weeks 2 and 4 indicating the presence of tight junction proteins within vascular walls. Scale bar: 100 µm. All data in this figure are presented as mean ± SEM of three independent experiments unless otherwise stated (^*^ *p* < 0.05, ^**^ *p* < 0.01, and ^***^ *p* < 0.001).

To determine if treatment with exogenous IGFBP7 to cranial bone defects would regulate the stability of vascular networks and enhance bone formation, a 1.8‐mm bilateral cranial bone defect^[^ ^23^ ^]^ was created in mice, followed by intravenous injection of IGFBP7 and IgG (control) at 1, 2, and 4 weeks post‐surgery. The results of micro‐vascular microcirculation (VCC) visualization and IF 4 days after IGFBP7/IgG treatment and micro‐CT and micro‐VCC visualization at 8 weeks post‐surgery to visualize the final outcomes of vascular regeneration and bone healing according to the specified grouping and drug administration schedule are depicted in Figure 3A.

The micro‐CT images showed that during the healing process of skull defects, IGFBP7 administration significantly promoted bone regeneration in the defect area as compared with the control group. There appeared to be a correlation with the timing of IGFBP7 administration, whereby a later administration resulted in an enhanced defect repair effect (Figure 3B,C). Notably, IGFBP7 administration at 4 weeks post‐surgery elicited the best outcome and the newly formed bone tissue exhibited normal morphology, with no signs of inflammation or abnormalities (Figure S4, Supporting Information). These results indicated that therapeutic intervention via IGFBP7 administration could significantly enhance bone defect healing.

To further elucidate if these results could be attributed to the stabilization of the vascular network, micro‐VCC analysis was performed 4 days after each injection (1, 2, and 4 weeks post‐surgery) to visualize the blood vessel network during the healing process. Compared with that observed in the control group, the vascular network in the IGFBP7‐treated defect area exhibited a more organized arrangement and clearer vessel demarcation (Figure S5, Supporting Information). Notably, IGFBP7 treatment reduced blood vessel density and increased vessel diameter index at each time point, with the most pronounced effect observed at 4 weeks post‐surgery. Micro‐VCC was performed again at 8 weeks post‐surgery to visualize the final outcome of the vessel network, and vessel‐masked maps were generated for quantitative analysis (Figure 3D). Additionally, flow velocity information was generated during micro‐VCC scanning. As expected, blood flow velocity of each IGFBP7 treatment group increased significantly at 8 weeks post‐surgery, indicating that IGFBP7 enhanced the perfusion of blood vessels and optimized the functionality of the fully formed vascular network (Figure 3E). Furthermore, blood vessel diameter showed significant increase at 8 weeks after IGFBP7 treatment. As shown in the masked maps, the diameters of the blood vessels became increasingly uniform (Figure 3F) following IGFBP7 administration at later time points (2 and 4 weeks). As irregular diameters may lead to impaired perfusion,^[^ ^24^ ^]^ the enlarged and well‐distributed diameters of the blood vessels signify that IGFBP7 significantly enhanced vascular network stability; the most profound effect was observed at 4 weeks post‐surgery. These results suggest that regularization of the blood vessel network led to gradual enlargement of the lumen and establishment of better blood flow. This indicates gradual stabilization of the blood vessels.

IF results showed high colocalization of ZO‐1 with CD31 in the vessels at the leading edge of defect healing, indicating that tight junction proteins within the blood vessel wall were enhanced by IGFBP7 treatment (Figure 3G). Overall, these results show that IGFBP7 treatment resulted in superior bone defect healing through fortification of tight junctions and promotion of blood vessel stabilization with the highest efficacy observed at week 4 of intervention with IGFBP7 treatment after surgery.

Thus, IGFBP7 delayed intervention enhances functional blood vessels and exhibits auxo action that improves vascular network stability and promotes bone defect healing. for enhancing stability of vascular networks to promote bone defect healing. Hence, IGFBP7 treatment may be effective as a therapeutic intervention for enhancing bone‐defect healing and stabilizing the vascular network by bolstering the tight junctions between ECs.

### 2.4 AT1001 (Tight Junction Regulator) at Relatively Later Stages Promotes Bone Defect Healing by Enhancing ZO‐1 Expression

To further determine whether known medications that enhance tight junctions (TJs) can elicit a similar impact as that of IGFBP7 on vascular stabilization and bone defect healing, we performed an in vivo assessment using AT1001 (also known as larazotide acetate), which is a synthetic peptide that functions directly as a TJ regulator in ECs.^[^ ^25^ ^]^

**Figure 4:**
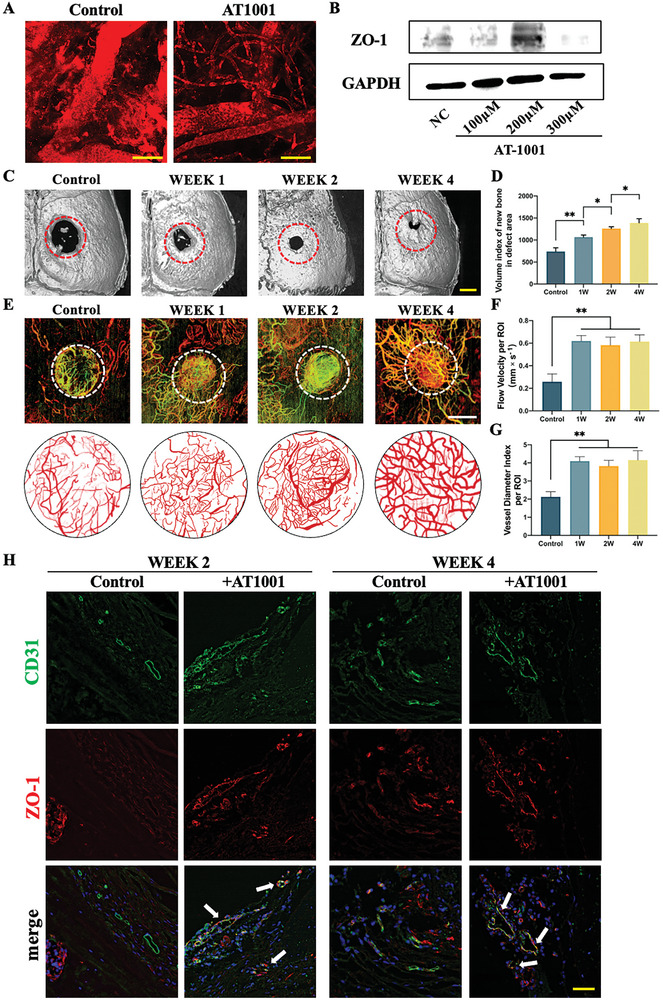
Tight junction regulator AT1001 promotes bone defect healing by enhancing ZO‐1 expression. A) Representative images of subcutaneous transplantation of HUVECs in nude mice with or without AT1001 treatment observed under a two‐photon microscope. Scale bar: 50 µm. B) Western blotting analysis showed that HUVECs exposed to 100, 200, and 300 µм of AT1001 showed up‐regulation of ZO‐1 expression. C) Representative micro‐CT 3D reconstruction images demonstrating regeneration of new bone at 8 weeks after cranial bone defect surgery. Scale bar: 1 mm. D) Relative volume index of new bone in the defect area was quantified using Inveon Research Workplace software and exhibited a positive correlation with the timing of AT1001 treatment; *n* = 6. E) Representative micro‐VCC scanning showed vessel networks within the cranial defect area more than 4 days after AT1001 treatment at different time points. Composite masked map of each image using Pyoct 7.0 software presented below each panel showing reduced blood vessel density and increased vessel diameter index varying with time. Scale bar: 600 µm. F) Flow velocity information per ROI generated in “FlowVel” mode and exported as number in unit mm s^−1^; *n* = 6. G) Vessel diameter index per ROI for each group in Figure E quantified using vResolve software; *n* = 6. H) Representative IF staining images for CD31 and ZO‐1 at weeks 2 and 4 indicating the presence of tight junction proteins within vascular wall. Scale bar: 100 µm. All data in this figure are presented as mean ± SEM of three independent experiments unless otherwise stated (^*^ *p* < 0.05, ^**^ *p* < 0.01, and ^***^ *p* < 0.001).

The therapeutic potential of AT1001 in regulating arteries has been previously tested in various acute and chronic inflammatory diseases^[^ ^26^, ^27^, ^28^ ^]^; however, its impact on neovascularity has rarely been reported. To confirm the impact of AT1001 on blood vessel wall permeability during neovascularization, we examined subcutaneous tumors in vivo and found that AT1001 effectively enhanced the integrity of neovascular walls and mitigated blood vessel leakage (Figure 4A; Figure S6, Supporting Information). Then, we assessed the effects of AT1001 on ZO‐1 expression in endothelial cells using western blotting analysis. We found that AT1001 treatment (100, 200, and 300 µм) promoted ZO‐1 expression on vascular endothelial cells, which improved the overall blood vessel wall integrity (Figure 4B). These results indicate that AT1001 effectively strengthens endothelial TJs, especially within the neovascular network, thereby diminishing blood vessel permeability and ultimately contributing significantly to the enhancement of vascular function.

To determine the probable effect of AT1001 on vascular network stability and bone formation, mice subjected to cranial bone defect surgery were stimulated with AT1001 and IgG (control) at 1, 2, and 4 weeks post‐surgery, identical to the timeline mentioned earlier. The micro‐CT results showed that AT1001 administration effectively promoted bone defect healing. Notably, administration at later time points produces better effects on bone defect repair (Figure 4C,D).

To further investigate its potential role in enhancing bone defect healing via the stabilization of blood vessels, we visualized the final blood vessel regeneration outcome using micro‐VCC. The findings showed that AT1001 treatment significantly increase blood vessel diameter and blood flow velocity at week 8 of IGFBP7 treatment. This enhanced blood vessel stability in the defect area, particularly during the final healing phase at 8 weeks (Figure 4E–G). This result further supports our hypothesis that AT1001 modulates bone defect healing by enhancing blood vessel stabilization.

To conclusively verify the in vivo mechanisms underlying AT1001‐mediated regulation of vascular stability and bone defect healing through ZO‐1 targeting, the forefront of the bone defect was analyzed using IF. The results showed that AT1001 treatment increased ZO‐1 co‐localization with CD31 within the defect region (Figure 4H). This implies that AT1001 enhances ZO‐1 expression in vascular endothelial cells within the bone defect area, thereby promoting vascular stability and bone healing.

These results clearly show that AT1001 effectively fortifies endothelial tight junctions, particularly within the neovascular network, and promotes bone defect healing by upregulating ZO‐1 expression, which confirms the feasibility of therapeutic interventional strategies based on the use of TJ regulators within the defect area to promote bone repair.

## 3 Discussion

Therapeutic angiogenesis, which promotes functional blood vessel formation to re‐establish adequate perfusion in ischemic tissues, is a promising treatment modality for bone defect healing.^[^ ^29^ ^]^ However, the critical role of vascular stabilization in facilitating bone‐defect healing remains poorly understood. In this study, we present for the first time an investigation of a new biological function of IGFBP7 in promoting blood vessel stabilization by upregulating ZO‐1 expression within endothelial tight junctions in vitro. Furthermore, our findings demonstrate the capacity of IGFBP7 to expedite bone defect healing while concurrently enhancing blood vessel stabilization in vivo, thereby providing continuous support for tissue repair. Thus, the findings of this study advance our knowledge of vascular stabilization and present a novel intervention target and treatment strategy for bone‐defect repair. Thus, this study introduces innovative concepts to the therapeutic landscape of tissue regeneration and repair.

Vascular permeability is a crucial parameter that affects the stability and integrity of blood vessels.^[^ ^30^ ^]^ In vivo and in vitro experiments have demonstrated that IGFBP7 significantly reduces the permeability of the vascular wall or cell monolayer, suggesting a role in promoting vascular stabilization. However, Komiya et al. reported contradictory findings. They showed that IGFBP7 administration at high concentrations (≥ 1000 ng mL^−1^ in vitro and in vivo) to ECs for a short duration (18 h) increased vascular permeability.^[^ ^31^ ^]^ In this study, we treated HUVECs with IGFBP7 at low concentration (160 ng mL^−1^) for an extended duration (48 h) and observed a significant inhibitory effect on the permeability of the HUVECs layer. This discrepancy in outcomes may be attributed to variations in the effective concentration of IGFBP7; nevertheless, the concentration used in the present study closely mirrors the physiological levels of IGFBP7 in adult serum (21–35 ng mL^−1^).^[^ ^32^ ^]^ Hence, we postulate that the regulatory effect of IGBFP7 on vascular integrity is concentration‐dependent, which suggests that vascular permeability reduces upon stimulation at concentrations approximating the physiological levels.

IGFBP7 is a secreted protein that has been implicated in regulating vessel remodeling and sprouting angiogenesis. Additionally, it plays a pivotal role in bone regeneration potential. This study focuses on the specific mechanism underlying IGFBP7‐mediated promotion of vascular stability through the upregulation of ZO‐1, which is a tight junction protein; however, the complex array of biological functions exhibited by IGFBP7 in various physiological processes cannot be ignored, and its broader effects on angiogenesis and neovascularization likely contribute to the overall efficacy of IGFBP7 in bone healing. In the context of neural signaling, IGFBP7 interacts with growth factors that are crucial for neuron development and survival in the peripheral nervous system. Furthermore, IGFBP7 promotes osteogenic differentiation of mesenchymal stem cells (MSCs), which enhances bone formation and cranial bone repair.^[^ ^33^, ^34^ ^]^ It regulates osteogenic differentiation through the Wnt/β‐catenin signaling pathway and acts as a negative regulator of RANKL‐induced osteoclastogenesis, both of which contribute to bone defect healing.^[^ ^35^ ^]^ Furthermore, IGFBP7 plays a notable role in bone regeneration potential involving the stimulation of bone formation in various models.^[^ ^33^, ^36^ ^]^ The enhancement of bone‐defect healing and stabilization of the vascular network observed in our study, can be attributed in part to the direct effect of IGFBP7 on osteogenic cells and the bone matrix. The intricate interplay between bone regeneration, angiogenesis, and skeletal nerve signaling highlights the importance of considering the multifaceted roles of IGFBP7 to gain a comprehensive understanding of its mechanisms of action.

Protein profiling performed in this study shows that IGFBP7 plays a significant role in regulating the membrane surface receptor IGF1R and the downstream PI3K/AKT signaling pathway. Previous studies have suggested that IGFBP7 may sustainably activate the downstream PI3K/AKT signaling pathway by prolonging the action of IGF1 on the IGF1R.^[^ ^37^ ^]^ However, other studies have used IGFBP7 at concentrations that are significantly higher than the physiological levels and observed that it inhibits IGF1R activation on adenocarcinoma cell surfaces, thereby suppressing the transmission of the downstream PI3K/AKT signaling pathway. The concentration used in our study was 160 ng mL^−1^, which closely approximates to the physiological levels. Therefore, this discrepancy may be attributed to variations in cell types and drug concentration. Moreover, our findings show that IGFBP7 directly upregulates IGF1R expression and activates the downstream PI3K/AKT signaling pathway in ECs, which highlights one possible intracellular mechanism underlying the regulation of vascular stability by IGFBP7.

Vascular development involves three key processes: formation (sprouting); stabilization and remodeling; regression. Over time, following the occurrence of bone defects, sprouting angiogenesis during the initial stages of angiogenesis is presumably facilitated by hypoxia, which upregulates expression of a number of genes involved in vessel formation and growth, providing the foundation for tissue regeneration by supplying essential nutrients, oxygen, and growth factors. As the tissue regeneration progresses, endogenous signals are required for negative feedback regulation to control excessive vascular sprouting. This, in turn, facilitates the attenuation of angiogenesis and initiates the vascular remodeling phase, ensuring that tissue regeneration proceeds in a controlled manner. During the subsequent stages of tissue regeneration and repair, vascular maturation approaches completion, and the vascular network attains stability, thereby optimizing its capacity for delivery of nutrients and molecules. In both our current and previous studies, we utilized IGFBP7 as a vascular stabilizer to intervene in the healing process of bone defects. We found that the best repair outcomes were achieved when the administration timeframe of the vascular stabilizers coincided with the phased demands of the tissue regeneration process. For instance, early therapeutic administration of IGFBP7 at 4 days post‐surgery inhibited angiogenesis and ultimately impaired bone defect repair;^[^ ^38^ ^]^ whereas delayed administration at 2–4 weeks post‐surgery significantly enhanced bone defect healing with peak efficacy observed at the 4‐week time point. These findings suggest that vascular stabilizers can be a viable option as novel drugs to promote bone defect repair and the timeframe of delivery is crucial to the outcomes.

Based on the fact that IGFBP7 specifically targets ZO‐1 and reinforces tight junctions, which leads to superior bone defect healing, we utilized the protease‐activated receptor 2 inhibitor AT1001, a well‐known medication that seals tight junctions by increasing ZO‐1 expression between endothelial cells,^[^ ^39^ ^]^ to further determine whether TJ regulators can elicit a similar effect on bone defect healing. Notably, our study revealed the pivotal role of AT1001 and other TJ regulators in possibly modulating the stability of the neovascular network and augmenting bone defect repair. Previous studies on TJ regulators have focused on their role in celiac and arteria‐related diseases.^[^ ^26^, ^27^, ^28^ ^]^ Yang et al. reported that AT1001 treatment stabilized the distribution and expression of ZO‐1 within cell–cell interfaces in both the ascending and descending aortas of β‐aminopropionitrile‐fed mice.^[^ ^25^ ^]^ In celiac disease, the barrier function is compromised, and patients with celiac disease patients exhibit enhanced intestinal permeability and disrupted TJs. Larazotide acetate, which is a crucial TJ regulator, mitigates this defect.^[^ ^40^ ^]^ Therefore, our study highlights the value of TJ regulators in clinical application for modulating the stability of neovascular networks. Furthermore, this inference hints at promising broader application prospects for TJ regulators in the field of tissue regeneration.

Nevertheless, this study has several limitations. Although our findings suggest the involvement of the IGF1R‐PI3K/AKT signaling pathway in mediating IGFBP7 signaling and promoting ZO‐1 expression, the precise mechanisms of underlying IGFBP7‐induced changes in intracellular signals, especially the interaction between IGFBP7 and IGF1R, need to be studied further. Although our study provides robust evidence for the involvement of the PI3K‐Akt pathway, we recognize that other signaling pathways such as VEGF and AMPK may also regulate ZO‐1 expression. These pathways represent promising avenues for future research to elucidate the complex molecular mechanisms underlying the effects of IGFBP7 on ZO‐1 expression. Furthermore, future studies on the recruitment of mural cells, generation of extracellular matrix, and specialization of the blood vessel wall are warranted to obtain a more comprehensive understanding of the process of vessel stabilization.

During the in vivo assays, IGFBP7 was systemically administered by intravenous injection. This necessitates a thorough validation of the effect of this mode of administration on overall bone mass within a systemic context. The limitations associated with the systemic administration of IGFBP7 need to be acknowledged, particularly with regard to the potential off‐target effects and short blood circulation time inherent to protein drugs. Despite these challenges, our experimental design was based on known biological functions and target specificity of IGFBP7. The dose and timing of IGFBP7 administration were optimized to minimize adverse effects. Furthermore, the translational potential of systemic administration, which closely mimics clinical applications, highlights the necessity of this approach. However, the specific pharmacokinetic behavior of IGFBP7, including its half‐life and interaction with other molecules in circulation, remains uncertain and requires further investigation. Although these findings provide a promising foundation for the systemic use of IGFBP7 in bone‐defect repair, further studies are warranted to completely characterize its pharmacokinetics, safety, and efficacy when administered intravenously.

In summary, we present a biological function of IGFBP7 that has not been previously described, which is the promotion of vascular stability by upregulating ZO‐1 expression within endothelial tight junctions in vitro. Our findings demonstrate the capacity of IGFBP7 to expedite bone defect healing while concurrently enhancing blood vessel stabilization in vivo and providing continuous support for tissue repair. Furthermore, treatment with the TJ regulator AT1001 effectively fortified endothelial tight junctions within the vascular network and promoted bone‐defect healing by upregulating ZO‐1 expression. Finally, we propose vascular stabilization as an innovative intervention target and treatment strategy for bone defect repair. This approach introduces novel concepts that significantly enrich the therapeutic landscape for tissue regeneration and repair.

## 4 Experimental Section

### Animals and Surgical Procedures

All animals used and surgical procedures performed in the present study were approved by the Institutional Animal Care and Use Committee of Peking University (approval number: LA2023209). In total, 60 male C57BL/CJ mice (8 weeks old) and 24 male BALB/c nude mice (4 weeks old) were purchased from Beijing HFK Bioscience Co. Ltd. (Beijing, China). To minimize potential confounding factors related to sex and the estrous cycle, only male animals were used in this study.

To investigate the effects of IGFBP7 or AT1001 on vessel permeability in vivo, nude mice (*n* = 6 per group) were subcutaneously injected with a mixture of human umbilical vein endothelial cells (1 × 10^7^; HUVECs) and Matrigel (0.1 mL; Becton, Dickinson and Company, Franklin Lakes, NJ, USA) at 4 °C on ice to construct subcutaneous tumors. One day after inoculation, the experimental group animals were injected with IGFBP7 (10 µg kg^−1^; 1334B7, R&D Systems, MN, USA) or AT1001 (50 mg kg^−1^ day^−1^; MCE, Monmouth Junction, NJ, USA), whereas the control mice were administered IgG. Then, 4 days after inoculation, FITC‐dextran (70 kDa, Invitrogen, Carlsbad, CA, USA) was constituted in 0.1 mL at a concentration of 10 mg m^−1^ and administered via intravenous tail injection. After 5 min, anesthesia was administered, and the mouse skin was peeled to expose the tumor. Vascular leakage images were observed using two‐photon confocal microscopy (Leica TCS SP8 DIVE, Heerbrug AG, Switzerland).

A cranial defect model was established to investigate the effects of IGFBP7 and AT1001 on vascular stability and bone defect healing. Before the surgical procedure, the mice were anesthetized with an intraperitoneal injection of pentobarbital sodium (100 mg kg^−1^). The cranial area of each mouse was exposed. A circular defect measuring ≈1.8 mm in diameter was created on both sides of the middle suture of the parietal region using a saline‐cooled round burr. A random number table was used to allocate the animals into different experimental categories. The subjects were initially divided into control and drug intervention groups. Subsequently, the drug intervention group was further stratification, and the animals were randomly divided into to one of three subgroups, each corresponding to a specific time point after surgical intervention: 1, 2, or 4 weeks post‐surgery. The sample size for each subgroup was standardized to six animals per group (N = 6). At each designated time point, a single intervention with IGFBP7 (10 µg kg^−1^) or AT1001 (50 mg kg^−1^ day^−1^) was administered via intravenous tail injection. After administering the course of drug treatment, the vascular morphology and bone condition were assessed.

### Two‐Photon Microscopy

After establishing subcutaneous tumors in the BALB/c nude mice, the skin was carefully removed to expose the tumor, and vascular leakage images were observed using a two‐photon confocal microscope (Leica TCS SP8 DIVE, Heerbrug, Switzerland). The parameters of the two‐photon microscope were as follows: scanning speed ranging from 2–8 ms pixel^−1^ (with a faster range of 2–5 ms) and zoom capability ranging from 1x–50x with a step size of 0.5x.

### Monitoring System of Vascular Microcirculation (Micro‐VCC) Scanning

Microvascular morphology alterations at the healing site of the bone defect were investigated at the microscopic level, using a monitoring system for micro‐VCC in vivo (OptoProbe Science LTD, England) 4 days post‐treatment with drugs or 8 weeks after defect modeling. The 3D OCT scanning range of the system was 10 ×10 ×2 mm, and the corresponding pixel data block size was 800 × 800 × 1280, meaning that each A‐scan contained 1280 pixels, each B‐scan contained 800 A‐scans, and each C‐scan contained 800 B‐scans. Microvascular imaging was performed without a contrast agent, with scanning repeated 20 times with each B‐scan. The 3D microvascular signals were extracted using the OCTA algorithm. Then, the collected data were extracted using vResolve software. Five A‐scans were performed at a single location, and the data from every five A‐scans were compared. A point with a blood flow signal generating a signal disturbance was identified as a blood vessel. The “FlowVel” scanning mode generated the flow velocity information and exported it in unit mm s^−1^. After signal extraction, the Reconstruction UI was used to generate the images. To minimize the interference of blood vessels in the carnal membrane on the newly formed blood vessels within the skull, this software was used to segment the images into three layers. Masked maps creation of vessel networks and analysis of ultra‐microscopic images was performed using Pyoct 7.0 software.

### Micro‐Computed Tomography (Micro‐CT) Scanning Evaluation

After 8 weeks of skull defect healing, the mice were euthanized using carbon dioxide. Subsequently, the skull tissue samples were extracted and fixed in a 4% (w/v) paraformaldehyde fixative at 4 °C for 24 h. Imaging data were acquired by scanning the tissue using a micro‐computed tomographic system (micro‐CT) (GANTRYSTD CT 3121; Siemens, Knoxville, TN). The parameters used for micro‐CT were: data set at 80 kV/500 µA; effective image pixel size of 33.658 µm; and exposure time of 1200 ms. Analysis of new bone formation within the tissue was performed using Inveon Research Workplace software 4.2, followed by 3D reconstruction of the skull tissue. The quantity of new bone was evaluated based on bone volume (BV).

### Immunofluorescence Staining for Skull Tissue

Following micro‐CT scanning, the skull tissue specimens were decalcified for 2 weeks with daily changes under standard protocols and subsequent paraffin coating. The paraffin‐embedded sections were rehydrated with xylene and ethanol. Following ddH_2_O rinsing, the sections were treated with a repair solution at high temperature or pressure.

IF staining of the bone tissue samples was performed according to the NEON multicolor fluorescent labeling system protocols (Jilin Histova Biotechnology, China). After three PBS washes, 3% (w/v) hydrogen peroxide was added to remove endogenous peroxidase, followed by staining. After three additional washes with PBS, the sections were treated with a sealing solution for 30 min to minimize nonspecific staining. Subsequently, the primary antibodies anti‐ZO‐1(ab307799; Abcam, London, UK) and anti‐CD31 (ab9498; Abcam, London, UK) were diluted in a suitable diluent and applied onto the tissue slice, which was placed overnight in a humidified chamber at 4 °C. The following day, after three rounds of PBS washing, the Pol HRP secondary antibody was added according to the primary antibody characteristics and incubated at room temperature for 30 min before washing again with PBS. The DendronFluor Branch Fluorescent TSA was diluted with the Fluoreffer Rapid Reaction solution to prepare a working solution, which was mixed and applied onto the slices. The reaction was allowed to occur at room temperature for 30–60 s. Subsequently, PBS was added to terminate the reaction, and the staining effect was observed using a fluorescence microscope. The antibody complex was removed from the sections prior to performing the next protein staining. A 2‐in‐1 buffer of AbCracker repair/Quick removal was prepared and heated in a microwave on high power until the solution boiled; then, the heat was turned off after 10 s. After 5 min, the slides were transferred to a water bath and cooled to room temperature before proceeding to the next round of antibody staining. Finally, after incubation with all polychromatic antibodies, the sections were washed with PBS for 5 min. Then, nuclear staining was performed at room temperature for 5 min using 4′,6‐Diamidino‐2‐phenylindole (DAPI; Sigma‐Aldrich, USA) solution. Once the sections were sealed, they were dried in a temperature‐controlled box at 37 °C for 30–60 min, observed, and analyzed for protein expression using a micro confocal microscope equipped with a Leica TCS SP8 STED camera (Heerbrug AG, Switzerland).

### Cell Culture

Human umbilical vein endothelial cells (HUVECs; Cat. No.100, ScienCell, Carlsbad, CA, USA) were cultured up to 6–8 passages according to the manufacturer's protocols in a humidified chamber with 5% CO_2_ at 37 °C.

### Endothelial Cell Layer Permeability Assay

A transwell kit (MCSP24H48, Millipore, Burlington, VT, USA) comprising upper and lower chambers, was used to assess the permeability of the single‐cell layer. For single‐cell layer models, the upper chamber of the 12‐well plate was covered with a bottom layer of Matrigel (Becton, Dickinson and Company, Franklin Lakes, NJ, USA) diluted with serum‐free medium at a ratio of 1:8. Then, a diluent (60 µL) was uniformly added, followed by incubation in an incubator at 37 °C for 1 h to polymerize the matrix glue into a film. The excess culture medium was removed after incubation, and the HUVECs were inoculated in the upper chamber at a cell density of 1 × 10^5^ cells/well. The experimental group was treated with IGFBP7 (160 ng mL^−1^; 1334B7, R&D Systems, MN, USA), whereas the control group was treated with an equal volume of complete medium, followed by incubation in a controlled environment at 37 °C and 5% CO_2_ for 48 h. Then, the upper chamber was washed twice with PBS. A new pore plate was used, and PBS (600 µL) was added. The new upper chamber was placed in the new pore plate, to which FITC‐dextran (100 µL; 70 kDa, Invitrogen, Carlsbad, CA, USA) solution was added. After incubation at 37 °C for 10, 30, 60, and 90 min, the optical density of PBS in the lower chamber hole plate was detected at 490 nm using an Enspire Microplate Reader (PerkinElmer, Waltham, Massachusetts, USA).

### Immunofluorescence Staining of In Vitro Cultured HUVECs and In Vivo Subcutaneous Hemangioma Vessels

HUVECs were cultured with IGFBP7 for 48 h and fixed with 4% (w/v) paraformaldehyde for 10 min to preserve their morphology and structure. After rinsing thrice with PBS, the cells were soaked in 3% (v/v) bovine serum albumin (BSA) solution for 30 min. The primary antibodies (ZO‐1 antibody: ab276131, Abcam, London, UK;IGF1R antibody: ab182408, Abcam, London, UK) were diluted and added to the cells, followed by overnight incubation at 4 °C. Subsequently, the excess primary antibody was thoroughly washed thrice with PBS, followed by the addition of secondary antibody dye (anti‐rabbit Alexa Fluor 555; Beyotime Biotechnology, Shanghai, China) and incubation in the dark at room temperature for 1 h. After washing again with PBS, diluted DAPI (Sigma–Aldrich, USA) was added to stain the cell nuclei, followed by incubation for 30 min. Finally, the samples were soaked in PBS. Imaging observations and image collection were performed using a laser confocal microscope (LSM710Carl Zeiss) with line scanning (0.00378Sx1000).

The spatial localization of ZO‐1 and CD31 in the endothelial cells of mouse subcutaneous tumors was assessed using IF staining. Briefly, the blood vessels were fixed with 4% (w/v) paraformaldehyde, followed by permeabilization with 0.1% (v/v) Triton X‐100 for 3 min. Subsequently, the membranes were blocked using 3% BSA. The vessel segments were incubated overnight with either anti‐ZO‐1 (ab276131, Abcam, London, UK) or anti‐CD31 antibody (ab9498; Abcam, London, UK) at 4 °C. After rinsing thrice with PBS, the vessel segments were incubated with Alexa Fluor 633‐conjugated goat anti‐rabbit secondary antibody (Thermo Fisher Scientific, Rochester, NY, USA) for 2 h in the dark at room temperature. The cell nuclei were counterstained with DAPI. Finally, the vessel segments were mounted on slides with the lumen side facing upward for confocal microscopy imaging (Leica TCS SP8 DIVE; Heerbrug, Switzerland).

### Western Blotting Analysis

The proteins were prepared and separated using SDS‐PAGE gel electrophoresis, followed by transfer onto a PVDF membrane (Millipore, USA). The membrane was blocked with 5% (w/v) skimmed milk powder. After washing, primary antibodies against ZO‐1 (ab276131; Abcam, London, UK), occludin (ab216327; Abcam), claudin‐5(ab131259; Abcam), IGF1R (ab182408; Abcam), PI3K/AKT(ab283852; Abcam), and p‐AKT(ab38449; Abcam) were added. This was followed by incubation with horseradish peroxidase‐labeled secondary antibody (A0208, Beyotime Biotechnology, Shanghai, China). After 1 h of incubation, TBST was added, and the cells were washed thrice. Finally, an ECL luminescence kit (Beijing CoWin Biotech Co., Ltd., Beijing, China) was used to detect specific antigens. ImageJ software (National Institute of Health, Bethesda, MD, USA) was used for analysis, and GAPDH monoclonal antibody(Beyotime Biotechnology, Shanghai, China) was used as the internal reference. The ratio of the grey value of the target protein to that of GAPDH was used to determine its relative expression.

### Quantitative Real‐Time Polymerase Chain Reaction (qRT‐PCR)

RNA was extracted using TRIzol reagent (Invitrogen, Carlsbad, CA, USA), followed by reverse transcription of total RNA into cDNA using a PCR thermal cycler (Takara Bio Inc., Tokyo, Japan). The primer sequences are listed in Table S1 (Supporting Information). Subsequently, quantitative real‐time polymerase chain reaction (qPCR) was performed on an ABI QuantStudio 3 Real‐Time PCR System (Applied Biosystems, Foster City, CA, USA) under the following conditions: initial denaturation at 95 °C for 1 min, followed by 40 cycles of denaturation at 95 °C for 20 s and annealing/extension at 60 °C for 1 min. The relative mRNA expression was determined using the 2^−∆∆Ct^ method. GAPDH was used as the internal reference.

### Liquid Chromatography–Mass Spectrometry (LC‐MS)

To investigate the effect of IGFBP7 treatment on protein expression in HUVECs, whole‐cell protein samples cultured for 48 h were extracted and subjected to quantitative proteomic analyses at the Peking University Health Science Center.

After protein quantification, cell protein extract (200 µg) from each group was subjected to enzymatic digestion using Filter‐Aided Sample Preparation (FASP). This was followed by separation and detection of unique peptides using liquid chromatography‐tandem mass spectrometry (LC‐MS/MS).

The digested peptides were analyzed using a Vanquish Neo coupled with an Orbitrap Exploreris 480 (Thermo Fisher Scientific) via LC‐MS/MS. Then, enzymatically cleaved peptide (500 ng) was injected onto a precolumn (100 µm × 2 cm, ReproSil‐Pur C18‐AQ, 3 µm) at a pressure of 800 bar and subsequently separated on an analytical column (75 µm × 15 cm, ReproSil‐Pur C18‐AQ, 3 µm) at a flow rate of 300 nL min^−1^. The chromatographic conditions were optimized using mobile phase A (0.1% formic acid (FA) in water) and B (0.1% FA in 80% acetonitrile). The gradient elution program was set at 90 min. The FAIMS DDA mode was used for mass spectrometry data acquisition. The primary mass spectrometry resolution was set at 60000 at m/z 200 with an automatic maximum injection time and target AGC of 300%. The scanning range was 350–1200. The secondary spectrum resolution was adjusted to 15 000 at m/z 200 with a scanning range of m/z 120–2000 and an isolation window of 1.6 Th. The target AGC was set to 75%, and the intensity threshold was set to 8e3. Full‐scan and multiple MS/MS spectra were acquired every 2 s. Dynamic exclusion time was set at 60 s. FAIMS compensation voltage was configured as −50 and −70 V, whereas other parameters remained at default settings.

The Proteome Discoverer 2.5 (Thermo Fisher Scientific) software SequestH search engine was used to query the SwissProt Human database. The mass error tolerance for the precursor ion in the primary spectrum was set at ±10 ppm, whereas that for fragment ions in the secondary spectrum was set at ±0.02 Da. Trypsin enzyme with full digestion or up to two missed cleavages was used. Additionally, alkylation of cysteine (C, +57.021 50 Da) was specified as a fixed modification, and oxidation of methionine (M, +15.994 92 Da) was set as a variable modification during data analysis. Percolator software was used to calculate the false discovery rate (FDR) of peptide‐spectrum matches (PSMs), ensuring that the false‐positive rates for PSMs, peptides, and proteins were controlled at 1%. For label‐free quantification, the Minora Feature Detector was used to extract chromatographic peaks, and the Feature Mapper aligned these peaks with the peptide identification results, allowing for a maximum retention time deviation of 10 min. Finally, the Precursor Quantifier tool was used to generate quantitative results, where protein intensity was determined as the sum of all peptide intensities.

### Flow Cytometric Analysis

A HUVEC suspension was obtained after trypsin treatment. The cells were fixed in 4% (w/v) paraformaldehyde at room temperature for 10 min. To detect the expression of intracellular proteins, the cells were treated with 0.25% (v/v) Triton X‐100 for 5 min. After blocking with 3% (w/v) BSA for 30 min, the cells were incubated with the anti‐IGF1R (intra) primary antibody (ab182408, Abcam, London, UK) diluted in 3% (w/v) bovine serum albumin (BSA). To assess the expression of the receptor on the cell membrane, the cells were blocked with BSA and incubated with the anti‐IGF1R (ab263907, Abcam, London, UK) primary antibody without using Triton X‐100. Then, the cells were washed thrice with PBS and incubated with secondary antibodies (Alexa Fluor‐488, ab150077; Abcam, London, UK) for 1 h at 24 °C. The cells were washed thrice with PBS and subjected to flow cytometric analysis using a NovoCyte Quanteon instrument (Agilent, Chengdu, China) and FlowJo software V10 (Tree Star, San Carlos, CA, USA).

### Statistical Analysis

All data are presented as the mean ± standard error of the mean (SEM). Statistical analyses were performed using the Prism software (version 9.0; San Diego, CA, USA). A two‐tailed unpaired Student's *t*‐test was used to compare different samples. For comparisons involving more than two sample groups, a one‐way unstacked analysis of variance (ANOVA) was used. Statistical significance was set at *p* < 0.05, whereas *p* ≤ 0.01 or 0.001 indicated a highly significant difference.

## Conflict of Interest

The authors declare no conflict of interest.

## Supporting information



Supporting Information

## Data Availability

Research data are not shared.
