# Supplementary material for: Delayed Administration of IGFBP7 Improved Bone Defect Healing via ZO‐1 Dependent Vessel Stabilization
Source: Adv Sci (Weinh). 2024 Dec 19;12(6):2406965. doi: 10.1002/advs.202406965 (PMC11809352; doi:10.1002/advs.202406965)
Supplement: Supplementary file 1 — Supporting Information [file ADVS-12-2406965-s001.docx]

**Supplementary material**

**Table S1** Primer sequences utilized for quantitative real‐time PCR analysis

| Target gene | | Forward sequence (5′–3′) | Reward sequence (5′–3′) |
| --- | --- | --- | --- |
| ZO-1 | AAGATCCAGCAATGAAGCC | | TGAGAAGTGGGTTTGGGA |
| GAPDH | TCCAAAATCAAGTGGGGCGA | | AAATGAGCCCCAGCCTTCTC |


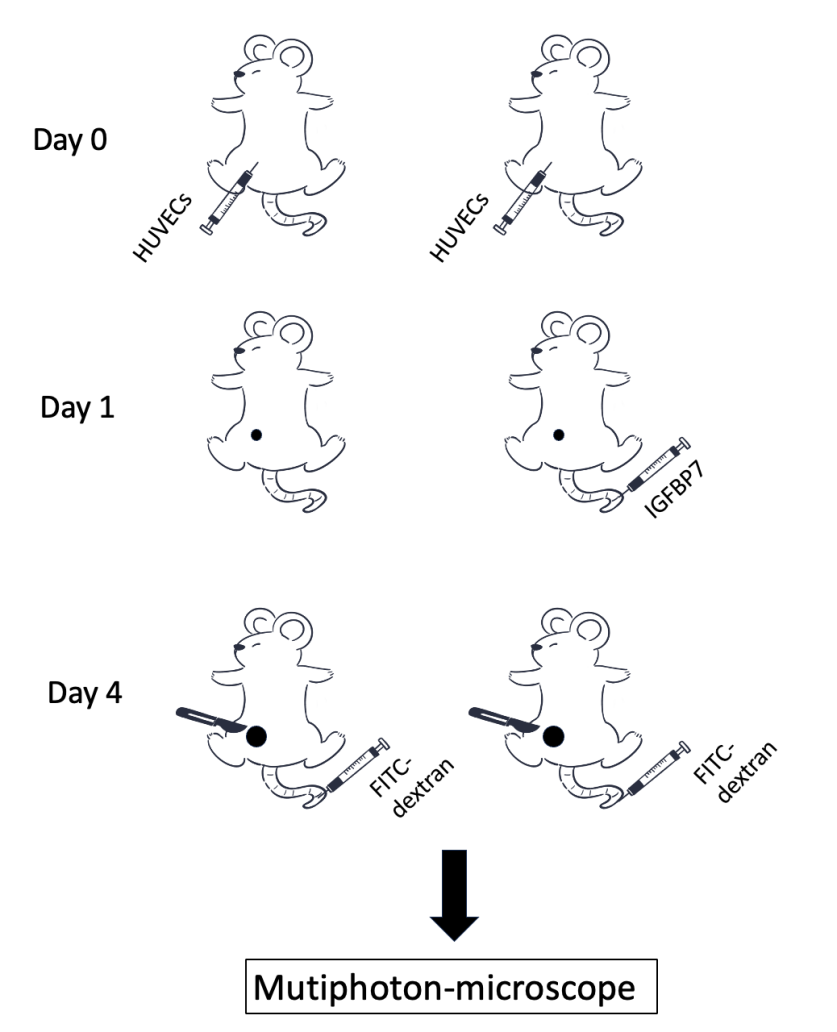


**Figure S1：Schematic illustration of subcutaneous hemangioma vessels and IGFBP7/IgG administration in nude mice.**

**
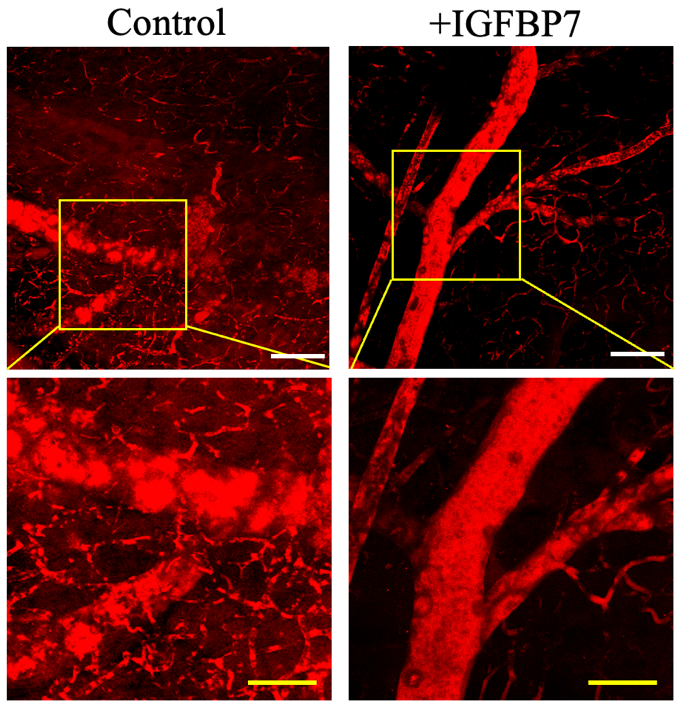
**

**Figure S2: Representative images of subcutaneous transplantation of HUVECs in nude mice with or without IGFBP7 (160 ng/mL) for 6 days, as observed under a two-photon microscope. White scale bar: 250 μm. Yellow scale bar: 100 μm.**


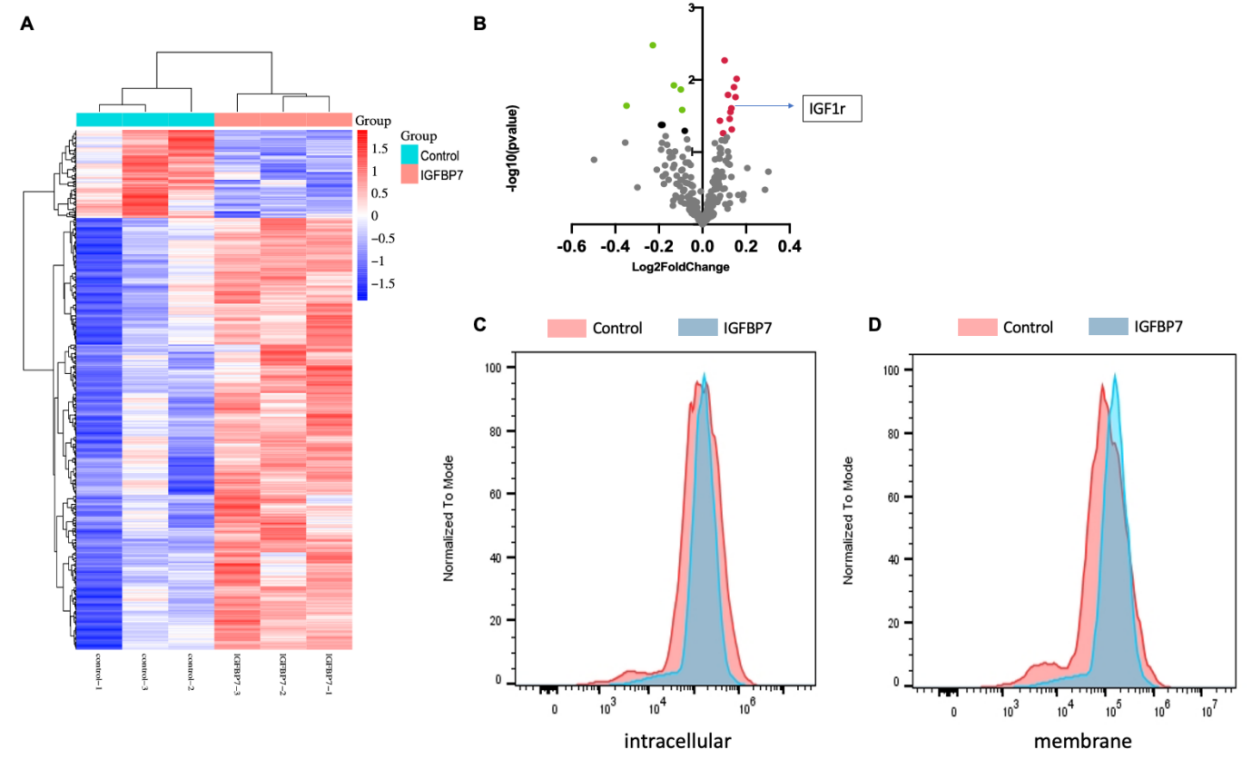


**Figure S3: The impact of IGFBP7 on differential protein expression in endothelial cells was analyzed using proteomics.**

(A) After 48 h of IGFBP7 stimulation at a concentration of 160ng/ml, the differential protein level clustering diagram was generated to compare the control group with the experimental group in endothelial cells. (B) Volcano plots showing the distribution of significance and fold changes of identified proteins in endothelial cells after 48 h of IGFBP7 treatment at a dose of 160ng/ml. The red and blue spots indicate significantly upregulated and downregulated proteins, respectively. (C) Protein flow cytometry analysis showed that IGFBP7 did not significantly affect intracellular IGF1R expression because the protein finger pattern of the IGFBP7 treatment group and control group overlapped. (D) Protein flow cytometry experiments showed that IGFBP7 promoted the expression of IGF1R on the cell membrane as evidenced by a partially separated protein finger pattern between the IGFBP7 treatment group and the control group.


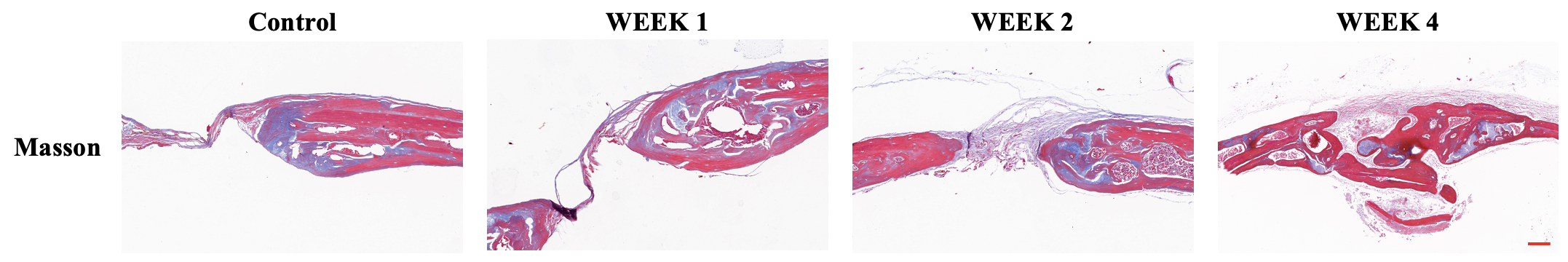


**Figure S4: Representative images of Masson's trichrome staining at the leading edge of bone defect healing after IGFBP7 treatment at different time points. Red scale bar: 200 μm.**


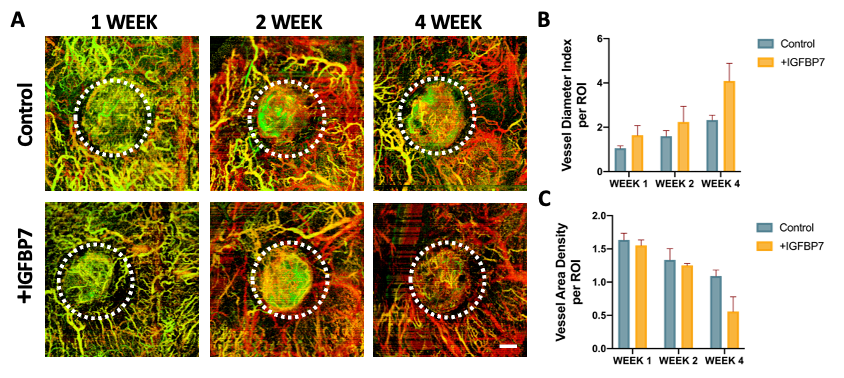


**Figure S5: Representative Micro-VCC scanning in cranial defect area 3-5 days after IGFBP7 stimulation at different time points.**

**(**A) Representative Micro-VCC scanning at 3-5 days after each injection (1 week/2 weeks/4 weeks post-surgery). As compared to the control group, the vascular network within the defect area treated with IGFBP7 exhibited more organized arrangement and clearer vessel demarcation. n=6. (B) The vessel diameter index per ROI in each group in Figure A were quantified using vResolve software. n=6. (C) The vessel area density per ROI in each group in Figure A were quantified using vResolve software. n=6.


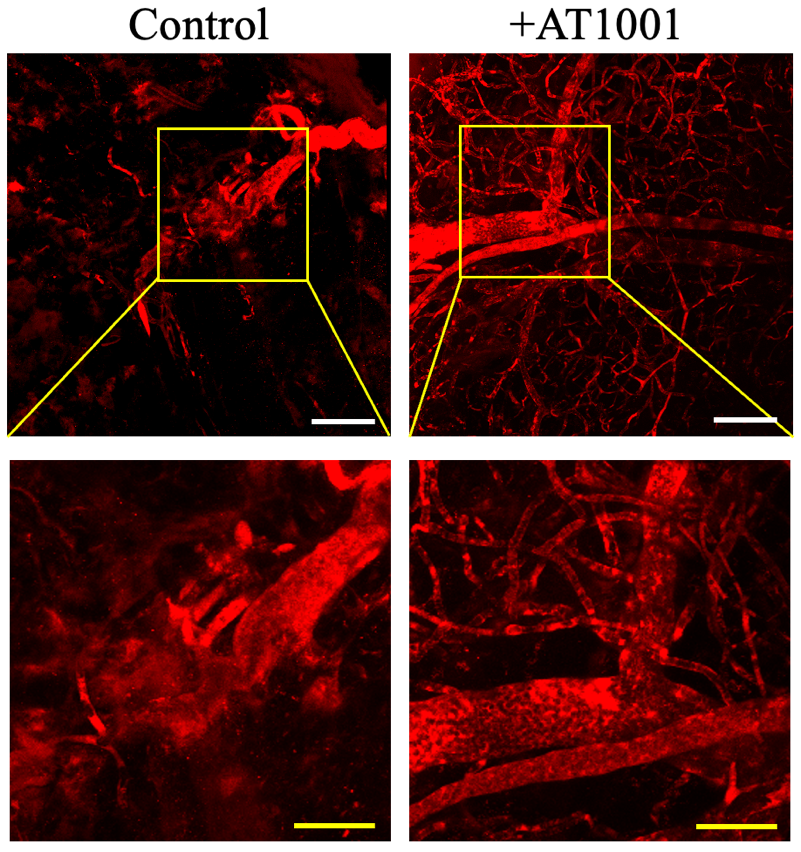


**Figure S6: Representative images of subcutaneous transplantation of HUVECs in nude mice with or without AT1001 treatment observed under a two-photon microscope. White scale bar: 250 μm. Yellow scale bar: 100 μm.**
